# Critical Roles for LIGHT and Its Receptors in Generating T Cell-Mediated Immunity during *Leishmania donovani* Infection

**DOI:** 10.1371/journal.ppat.1002279

**Published:** 2011-10-06

**Authors:** Amanda C. Stanley, Fabian de Labastida Rivera, Ashraful Haque, Meru Sheel, Yonghong Zhou, Fiona H. Amante, Patrick T. Bunn, Louise M. Randall, Klaus Pfeffer, Stefanie Scheu, Michael J. Hickey, Bernadette M. Saunders, Carl Ware, Geoff R. Hill, Koji Tamada, Paul M. Kaye, Christian R. Engwerda

**Affiliations:** 1 Queensland Institute of Medical Research and the Australian Centre for Vaccine Development, Herston, Queensland, Australia; 2 Institute for Molecular Biology, University of Queensland, St Lucia, Queensland, Australia; 3 Department of Pathobiology, School of Veterinary Sciences, University of Pennsylvania, Philadelphia, Pennsylvania, United States of America; 4 Institute of Medical Microbiology and Hospital Hygiene, University of Duesseldorf, Duesseldorf, Germany; 5 Centre for Inflammatory Diseases, Monash University, Department of Medicine, Monash Medical Centre, Clayton, Victoria, Australia; 6 Centenary Institute, Newtown, New South Wales, Australia; 7 Infectious and Inflammatory Diseases Centre, Sanford|Burnham Medical Research Institute, La Jolla, California, United States of America; 8 Marlene and Stewart Greenebaum Cancer Center, University of Maryland, Baltimore, Maryland, Unites States of America; 9 Hull York Medical School, Department of Biology, York University, York, United Kingdom; National Institute of Health, United States of America

## Abstract

LIGHT (TNFSF14) is a member of the TNF superfamily involved in inflammation and defence against infection. LIGHT signals via two cell-bound receptors; herpes virus entry mediator (HVEM) and lymphotoxin-beta receptor (LTβR). We found that LIGHT is critical for control of hepatic parasite growth in mice with visceral leishmaniasis (VL) caused by infection with the protozoan parasite *Leishmania donovani*. LIGHT-HVEM signalling is essential for early dendritic cell IL-12/IL-23p40 production, and the generation of IFNγ- and TNF-producing T cells that control hepatic infection. However, we also discovered that LIGHT-LTβR interactions suppress anti-parasitic immunity in the liver in the first 7 days of infection by mechanisms that restrict both CD4^+^ T cell function and TNF-dependent microbicidal mechanisms. Thus, we have identified distinct roles for LIGHT in infection, and show that manipulation of interactions between LIGHT and its receptors may be used for therapeutic advantage.

## Author Summary

Visceral leishmaniasis (VL) is a potentially fatal human disease caused by the intracellular protozoan parasites *Leishmania donovani* and *L. infantum* (*chagasi*). Parasites infect macrophages throughout the viscera, though the spleen and liver are the major sites of disease. VL is responsible for significant morbidity and mortality in the developing world, particularly in India, Sudan, Nepal, Bangladesh and Brazil. Because of the intrusive techniques required to analyse tissue in VL patients, our current understanding of the host immune response during VL largely derives from studies performed in genetically susceptible mice. We have discovered that mice which are unable to produce a cytokine called LIGHT have poor control of *L. donovani* infection in the liver, compared with wild-type control animals. In addition, we demonstrated that LIGHT has distinct roles during VL, depending on which of its two major cell-bound receptors it engages. Finally, we identified an antibody that stimulates the lymphotoxin β receptor (one of the LIGHT receptors), that can stimulate anti-parasitic activity during an established infection, thereby identifying this receptor as a therapeutic target during disease.

## Introduction

Tumour necrosis factor (TNF) superfamily members are involved in many biological functions, including cell growth and differentiation, apoptosis and organogenesis [1]. This broad range of activities is achieved by TNF family members interacting with functional receptors associated with distinct cell signalling pathways [2]. TNF, lymphotoxin (LT)α, LTβ and LIGHT (TNFSF14) comprise a closely related set of ligands in the TNF family [3], [4]. TNF exists as a cell-bound or soluble homotrimer that binds TNF receptor (TNFR)1 and TNFR2 [5], [6]. LTα can form a soluble homotrimer (LTα_3_) that binds TNFR1, TNFR2 and HVEM [5], [7], but can also form a cell-bound heterotrimer with LTβ (LTα_1_β_2_) that binds and signals through LTβR [8]. LIGHT exists in cell-bound and soluble forms that interact with both LTβR and herpes virus entry mediator (HVEM) [7], [9], [10]. HVEM also engages members of the immunoglobulin superfamily; B and T lymphocyte attenuator (BTLA) [11] and CD160 [12], as well as the envelope glycoprotein D of Herpes Simplex virus [13]. HVEM activates BTLA inhibitory signalling via SHP phosphatases suppressing T cell activation [14]. LIGHT, LTα and the Ig superfamily ligands can also activate HVEM-dependent cell survival signalling via NF-κB [15].

LIGHT has emerged as a key mediator of inflammation and immune homeostasis [4], [14]. There is broad expression of LIGHT and HVEM in the hematopoietic compartment [7], [9], [16], [17], [18], while LTβR expression is largely restricted to stromal and myeloid cells [7], [19], [20]. LTβR and HVEM are implicated as key host defence mechanisms against persistent viral [21] and bacterial pathogens [22]. However, little is known about the role of these receptors in infection with parasites that establish persistent infections in their hosts.

The protozoan parasite *Leishmania donovani* causes persistent infections in humans and experimental animals [23], [24]. We and others have defined important roles for TNF and LTα in host resistance in a mouse model of visceral leishmaniasis (VL) caused by *L. donovani* [25], [26], [27]. This disease model is characterised by an acute, resolving infection in the liver involving the formation of pro-inflammatory granulomas around infected Kupffer cells, and the establishment of a chronic infection in the spleen (reviewed in [24], [28], [29], [30]). Mice deficient in TNF are highly susceptible to *L. donovani* infection, and die in the second month of infection with unchecked parasite growth [25], [26], [31]. However, TNF also induces disease pathology in the spleen, including the loss of marginal zone macrophages and down-regulation of chemokine receptor expression by dendritic cells (DCs) [31], [32]. Mice lacking LTα display a less severe phenotype characterised by disrupted cellular trafficking into the liver and reduced control of hepatic parasite growth, although ultimately, infection is resolved in this organ [26].

Here we investigated the impact of *L. donovani* infection in LIGHT-deficient mice, as well as the roles of LIGHT binding each of its functional, cognate receptors during infection. We report a critical role for LIGHT in the resolution of hepatic infection, and more specifically, identify an important role for LIGHT-HVEM interactions in stimulating IL-12 production by DCs, and hence in the control of parasitic infections. Conversely, we also discovered that blockade of LIGHT-LTβR interactions dramatically enhanced early anti-parasitic immunity. Thus, we have identified distinct and opposing roles for LIGHT engagement of each of its receptors during infection.

## Results

### Organ-specific expression of LIGHT in response to L. donovani infection

**Figure 1:**
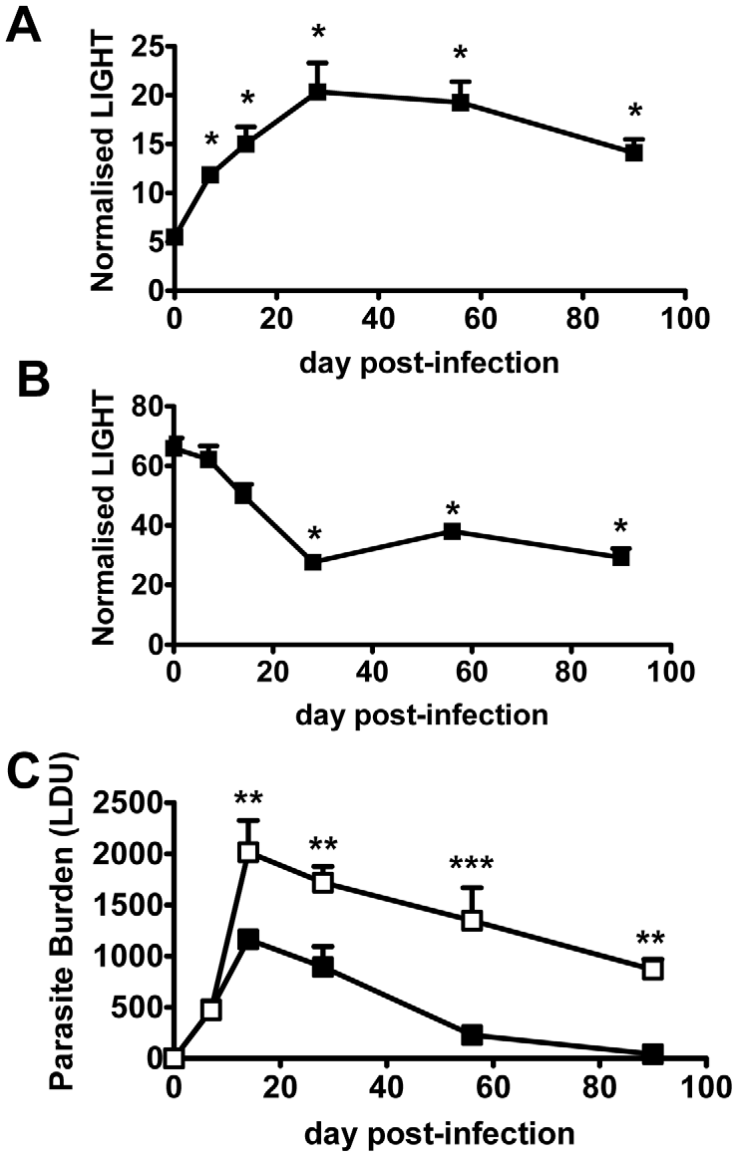
LIGHT is required for efficient parasite clearance in the liver. C57BL/6 mice were infected with L. donovani, and LIGHT mRNA accumulation was measured in liver (A) and spleen (B) at indicated times (expressed as the number of LIGHT mRNA molecules per 1000 HPRT mRNA molecules). (C) C57BL/6 (closed squares) and B6.LIGHT^−/−^ (open squares) mice were infected with *L. donovani*, and hepatic parasite burdens were measured from day 7 p.i. to day 90 p.i. and data represent the mean +/−SEM. One representative experiment of at least 2 performed is shown (n = 4–5 mice per treatment group in each experiment). Statistical differences of p<0.05 (*), p<0.01 (**) or p<0.001 (***) are indicated.

Homeostatic levels of LIGHT mRNA in liver (Figure 1A) and spleen (Figure 1B) differed by an order of magnitude in naïve mice. Following *L. donovani* infection, LIGHT mRNA accumulation increased in the liver over the first 28 days, and remained elevated despite infection largely resolving (Figure 1C). In contrast, the initially high splenic LIGHT mRNA levels decreased over the first 28 days of infection (Figure 1B), and remained diminished as a persistent *L. donovani* infection became established (Figure S1A). Thus, an organ-specific pattern of LIGHT mRNA expression emerged in response to *L. donovani* infection.

To establish whether LIGHT was required to control infection, we infected LIGHT-deficient and control C57BL/6 mice with *L. donovani* and followed the course of infection in the spleen and liver for 90 days. Despite no difference in hepatic parasite burdens in the first 7 days of infection, parasite growth was significantly greater in the livers of LIGHT-deficient mice from day 14 p.i. onwards. Furthermore, these mice failed to fully resolve hepatic infection in the time period studied (Figure 1C). TNF, IFNγ and nitric oxide (measured as the surrogate marker inducible nitric oxide synthase; NOS2) are all critical for control of *L. donovani* in the liver [26], [27], [31], [33], [34]. Serum TNF and IFNγ levels were reduced, and the accumulation of hepatic NOS2, IFNγ and TNF mRNA were all lower in LIGHT-deficient mice at 14 days, compared with control animals (Figure S1B–E). In the spleen, there were no significant differences in parasite burdens between C57BL/6 and B6.LIGHT^−/−^ mice at any time point studied (data not shown). The accumulation of NOS2 mRNA was much lower in the spleen of C57BL/6 and B6.LIGHT^−/−^ mice at day 14 p.i., compared with the liver, and no difference in IFNγ, TNF and NOS2 mRNA accumulation in the spleen between mouse strains was observed at this time point (Figure S1B–E). We therefore focused our attention on the liver.

**Figure 2:**
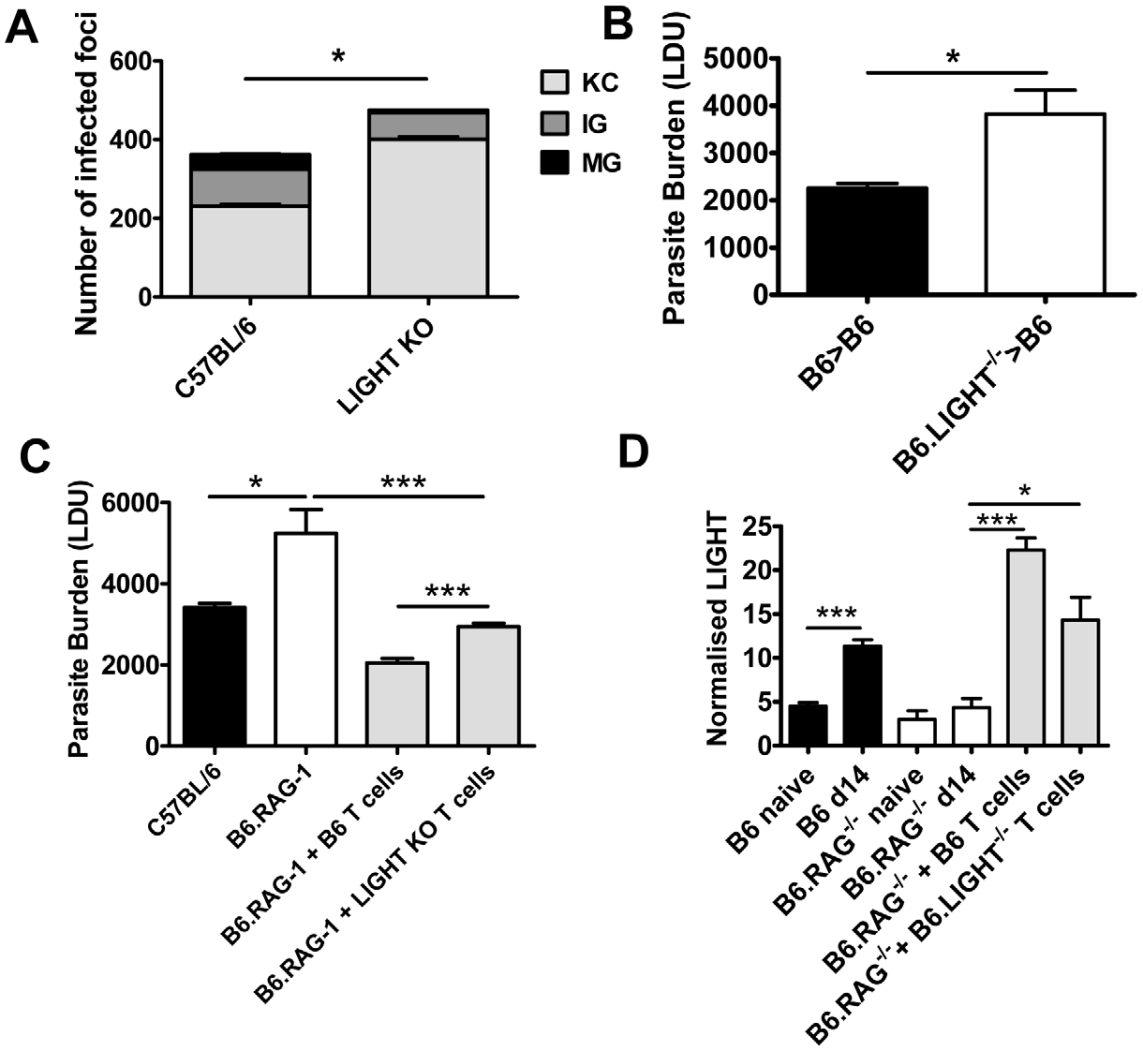
LIGHT from hematopoietic cells is required for efficient granuloma formation and control of *L. donovani* in the liver. The number and maturity of hepatic granulomas were evaluated on liver sections taken at day 14 p.i. from C57BL/6 and B6.LIGHT^−/−^ mice, as indicated (A), and data represent the mean frequency of infected Kupffer cells (KC), immature granulomas (IG) and mature granulomas (MG) per liver +/−SEM. (B) C57BL/6 mice engrafted with BM from C57BL/6 mice (B6>B6) (closed bars) and B6.LIGHT^−/−^ mice (B6.LIGHT^−/−^>B6) (open bars) were infected with *L. donovani* and hepatic parasite burdens were measured at day 14 p.i. and are represented as the mean +/−SEM. Parasite burden (C) and LIGHT mRNA accumulation (D) in the livers of C57BL/6 and B6.RAG1^−/−^ mice (plus or minus C57BL/6 or LIGHT-deficient CD4^+^ T cells and CD8^+^ T cells (equal numbers), as indicated) at day 14 p.i.. One representative experiment of at least 2 performed is shown (n = 4–5 mice per treatment group in each experiment). Statistical differences of p<0.05 (*), p<0.01 (**) or p<0.001 (***) are indicated.

The formation of pro-inflammatory granulomas around infected Kupffer cells is a critical step in host control of parasite growth in the liver [24], [28], [29], [30]. Liver immunohistochemistry revealed an increased number of inflammatory foci associated with increased parasite burden and impaired formation of inflammatory granulomas in B6.LIGHT^−/−^ mice at day 14 p.i., relative to control mice, as indicated by a greater frequency of infected Kupffer cells with no surrounding leukocytes (KC), and a lower frequency of immature (IG) and mature granulomas (MG) (Figure 2A). To ensure that the failure to develop anti-parasitic immunity in the liver did not result from an as yet unidentified developmental defect in LIGHT-deficient mice, BM chimeras were made by engrafting LIGHT-deficient or control (C57BL/6) BM cells into lethally irradiated C57BL/6 mice. These mice were infected and parasite burdens measured 14 days later. BM chimeric mice responded to hepatic infection in accordance with their source of BM (Figure 2B). Hepatic parasite burdens were significantly increased in LIGHT-deficient BM chimeras compared to controls, indicating that LIGHT production by leukocytes was required for the efficient generation of anti-parasitic immunity in the liver at this early time point in infection. Additional experiments in T and B cell-deficient B6.RAG1^−/−^ mice receiving LIGHT-deficient or wild-type T cells showed that LIGHT production by T cells was not required for the development of anti-parasitic immunity in the liver during the first 14 days of infection (Figure 2C). However, we cannot exclude a role for T cell-derived LIGHT in the generation of optimal early host immunity following *L. donovani* infection because we did find a small, but significant difference (p<0.001) in parasite burden at day 14 p.i. between B6.RAG1^−/−^ mice that received LIGHT-deficient T cells and those that received wild-type T cells (Figure 2C). We also found that T cells *per se* were not a major source of hepatic LIGHT mRNA, although their presence was required for LIGHT expression to increase in the liver during this early period of infection (Figure 2D). Together, these data indicate that the generation of immune responses against L. donovani in the liver were impaired in the first 14 days of infection the absence of LIGHT.

### Treatment with anti-HVEM mAb impairs parasite clearance while treatment with anti-LTβR mAb promotes this process

**Figure 3:**
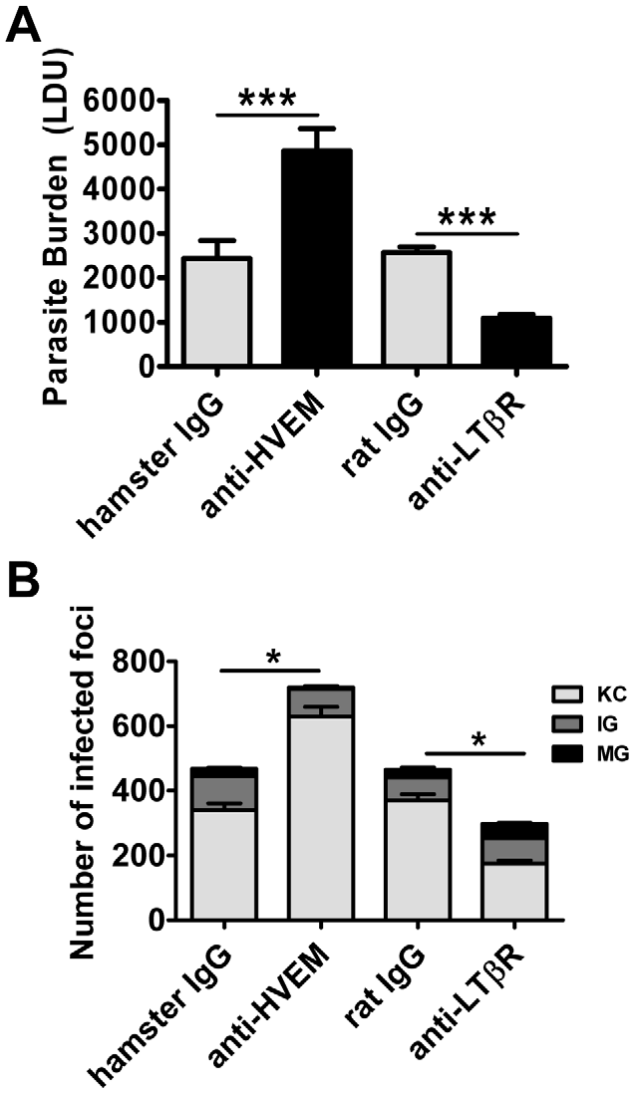
Distinct effects of blocking LIGHT-HVEM and LIGHT-LTβR interactions on parasite growth in the liver. Parasite burdens were determined in the livers of L. donovani infected mice treated with anti-HVEM (LH1) mAb or control hamster IgG or anti-LTβR (LLBT2) mAb or control rat IgG (A). Data are represented as the mean +/− SEM at day 14 p.i.. The number and maturity of hepatic granulomas (B) were measured at day 14 p.i., as described in the legend of Figure 2. One representative experiment of 3 performed is shown (n = 5 mice per treatment group in each experiment). Statistical differences of p<0.05 (*) or p<0.001 (***) for control versus mAb-treated mice are shown.

Treatment with anti-HVEM mAb (LH1) that blocks LIGHT binding to HVEM, but not HVEM-BTLA interactions [35], significantly increased hepatic parasite load at day 14 p.i. in mice, similar to the increase in parasite burden observed in LIGHT-deficient mice (Figure 3A). Surprisingly, hepatic parasite burdens were significantly decreased by treatment of mice with anti-LTβR mAb (LLBT2) that blocks LIGHT binding to LTβR, but not LTα_1_β_2_-LTβR interactions [35] (Figure 3A). Antibody treatments had no significant effect on the low splenic parasite burden at this time point (Figure S2). The formation of granulomas was significantly impaired by anti-HVEM mAb, as indicated by a greater frequency of KC and a lower frequency of IG and MG (p<0.05, κ^2^ analysis; Figure 3B). In contrast, granuloma formation was significantly enhanced by anti-LTβR mAb, as indicated by a lower frequency of KC and a higher frequency of IG and MG (p<0.05, κ^2^ analysis; Figure 3B). Thus, these results indicate that HVEM and LTβR have distinct and opposing roles during the first 14 days of infection.

### Stimulation of LTβR improves parasite clearance during an established infection

**Figure 4:**
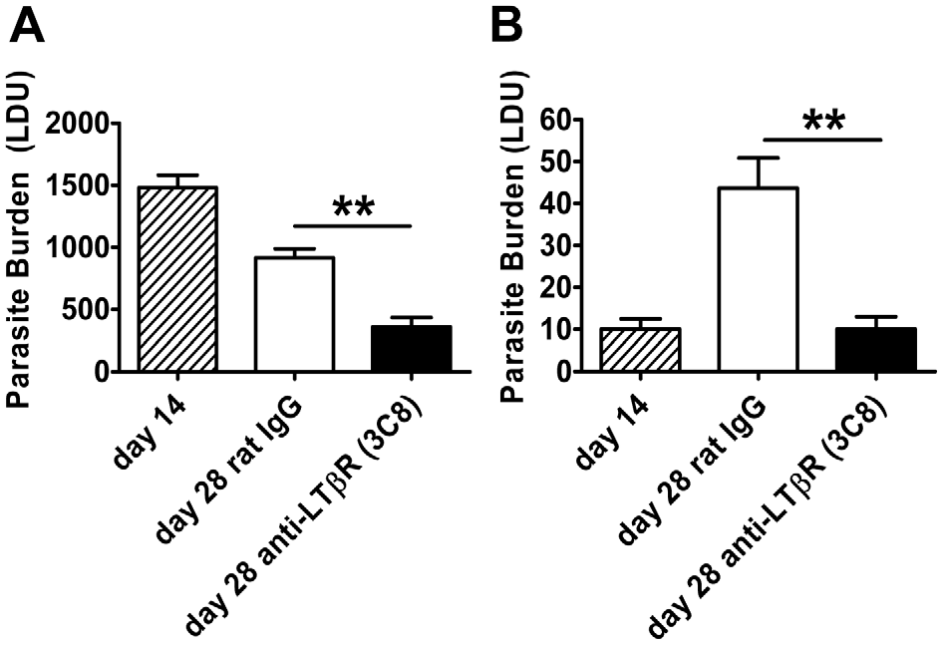
Agonistic stimulation of LTβR only enhances parasite clearance in an established infection. C57BL/6 mice with an established L. donovani infection were treated with control rat IgG or agonistic anti-LTβR mAb (3C8) from day 14 p.i. onwards and parasite burdens were determined in the liver (A) and spleen (B) at day 28 p.i.. One representative experiment of two performed is shown (n = 5 mice per treatment group in each experiment). Statistical differences of p<0.01 (**) for control versus treated mice are shown.

To further investigate the role of LTβR in VL, we treated mice with the agonist anti-LTβR antibody (3C8) which blocks binding of both LTα_1_β_2_ and LIGHT to LTβR, yet functions as an agonist directly activating LTβR signalling pathways [36], [37]. The anti-LTβR 3C8 enhanced parasite clearance in the liver during an established infection (Figure 4A), but had no anti-parasitic effect in the first 14 days of infection (data not shown), unlike the anti-LTβR mAb LLBT2 (Figure 3A). Importantly, 3C8 also prevented parasite growth in the spleen between days 14 to 28 p.i. (Figure 4B). In contrast, treatment with LLTB2 during established infection (days 14–28 p.i.) had no effect on parasite clearance in the liver or spleen (data not shown). Thus, treatment of *L. donovani*-infected mice with two different anti-LTβR mAbs had distinct effects on the course of infection, reflecting different functional properties of these mAbs.

### Treatment with anti-HVEM mAb impairs Th1-mediated immunity

**Figure 5:**
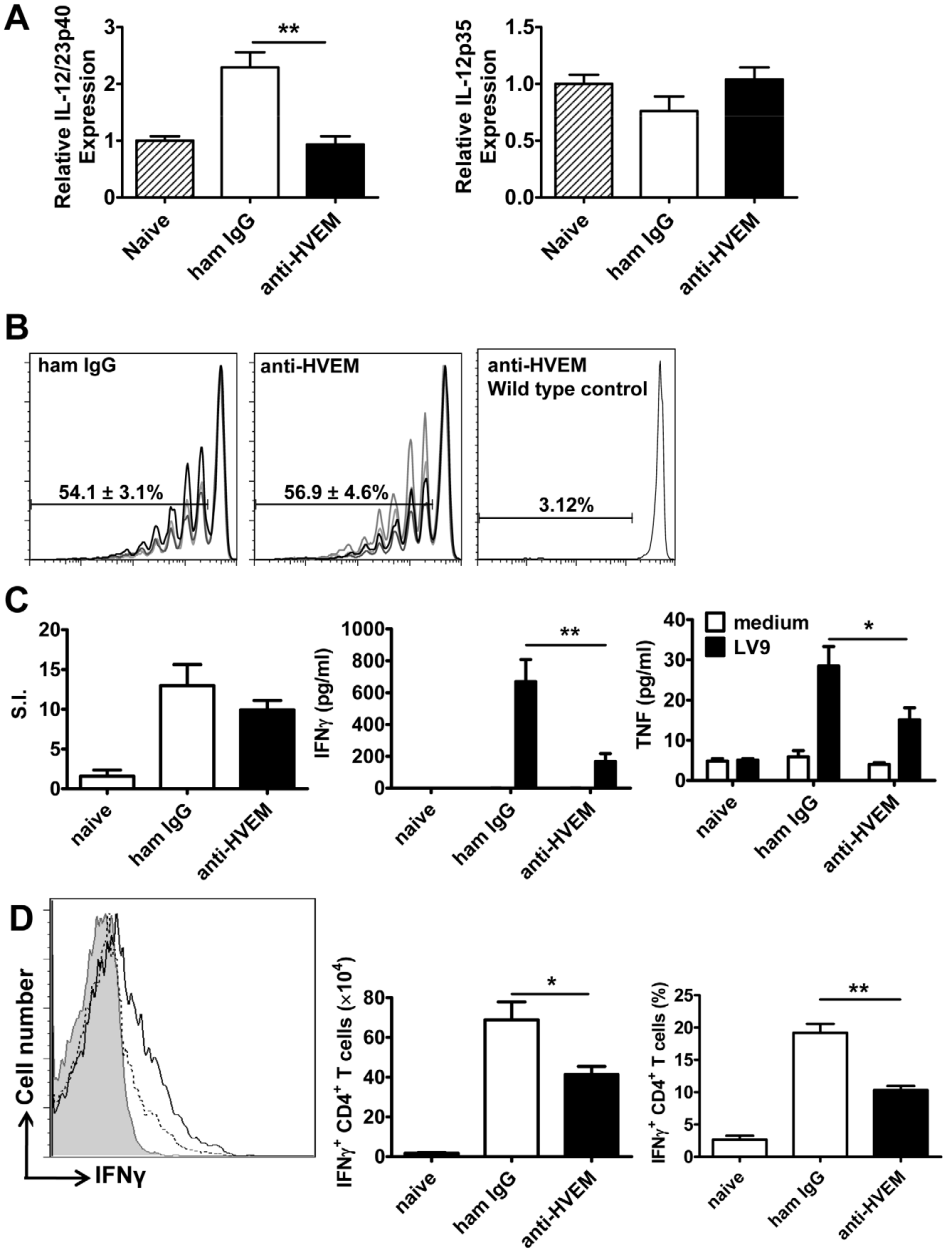
LIGHT-HVEM interactions are required for early DC IL-12/IL-23p40 mRNA accumulation, and antigen-specific CD4^+^ T cell IFNγ and TNF production. C57BL/6 mice were treated with anti-HVEM mAb or control hamster IgG or left untreated prior to infection with 1×10^8^ L. donovani amastigotes. IL-12/IL-23p40 mRNA accumulation was measured in splenic CD11c^+^ DC that had been enriched by positive selection by MACS from naïve mice (hatched bars), untreated mice (grey bars), hamster IgG treated mice (open bars) or anti-HVEM treated mice (closed bars) at 5 hours p.i. (A). C57BL/6 mice treated with control hamster IgG or anti-HVEM mAb received CD45.1^+^ CFSE-labelled OVA-specific CD4^+^ T cells (OT-II) prior to infection with 2×10^7^ OVA-expressing L. donovani. OT-II CFSE dilution in the spleen was assessed by flow cytometry on day 4 p.i., after gating on CD45.1^+^CD4^+^TCRβ^+^ cells (B) and % CFSE dilution +/− SEM is shown on each plot (CFSE dilution for 4 individual mice in each treatment group is shown, as well as CSFE dilution in a control animal that was infected with wild type parasites). Splenic CD4^+^ T cells were purified on day 14 p.i. from naïve mice or L. donovani-infected mice treated with control hamster IgG (open bars) or anti-HVEM mAb (closed bars) by positive MACS selection. CD4^+^ T cells were co-cultured with paraformaldehyde-fixed L. donovani amastigotes and irradiated spleen cells. Antigen-specific CD4^+^ T cell proliferation, as well as IFNγ and TNF levels in cell culture supernatants were measured by cytometric bead array at 72 h post-stimulation (C). Hepatic CD4^+^ T cell (CD4^+^TCRβ^+^) IFNγ production was measured in L. donovani-infected mice treated with either control hamster IgG (solid line) or anti-HVEM (dashed line) mAb on day 14 p.i.. Representative histograms showing IFNγ production by gated CD4^+^ TCRβ^+^ cells are shown and compared to isotype control antibody (solid gray) (D). Both the total number and frequency of IFNγ-producing CD4^+^ T cells are shown graphically. One representative experiment of 3 performed with similar outcome is shown (n = 4 mice per treatment group in each experiment). Statistical differences of p<0.05 (*), p<0.01 (**) or p<0.001 (***) for control versus mAb-treated mice are shown.

We next sought to identify anti-parasitic mechanisms dependent upon LIGHT-HVEM signalling. We previously showed that early splenic IL-12/IL-23p40 production by DC is critical for the efficient generation of immunity in the liver [38], [39]. Although no change in IL-12p35 mRNA accumulation was observed in any treatment group, the anti-HVEM mAb (LH1) inhibited splenic DC IL-12/IL-23p40 mRNA accumulation in response to L. donovani infection (Figure 5A). We next evaluated the importance of LIGHT-HVEM co-stimulatory signals for the development of L. donovani-specific CD4^+^ T cell priming and Th1 differentiation, the latter being a known IL-12-dependent process [39], [40], [41]. Mice were injected with CFSE-labelled OVA-specific CD4^+^ (OT-II) T cells, then infected with transgenic OVA-expressing L. donovani [42], and antigen-specific CD4^+^ T cell proliferation was assessed 4 days later by CFSE dilution. No antigen-specific CD4^+^ T cell proliferation was observed when mice were infected with wild-type parasites (Figure 5B), so no bystander activation had occurred, and OT-II cell proliferation occurred equally in control and anti-HVEM-treated mice (Figure 5B), indicating that LIGHT-HVEM interactions were not required for early priming of CD4^+^ T cell proliferation. In support of this result, proliferation of polyclonal antigen-specific, CD4^+^ T cells was similar between cells isolated from the spleens of control-treated and anti-HVEM treated mice (Figure 5C). However, production of IFNγ and TNF by these antigen-specific CD4^+^ T cells was inhibited by anti-HVEM mAb (Figure 5C). Furthermore, direct *ex vivo* production of IFNγ by hepatic CD4^+^ T cells (both total number and frequency) was significantly reduced by anti-HVEM mAb (Figure 5D), indicating that LIGHT-HVEM interactions play an important role in generating Th1 cell responses following *L. donovani* infection.

### Treatment with anti- LTβR mAb promotes Th1-mediated immunity

**Figure 6:**
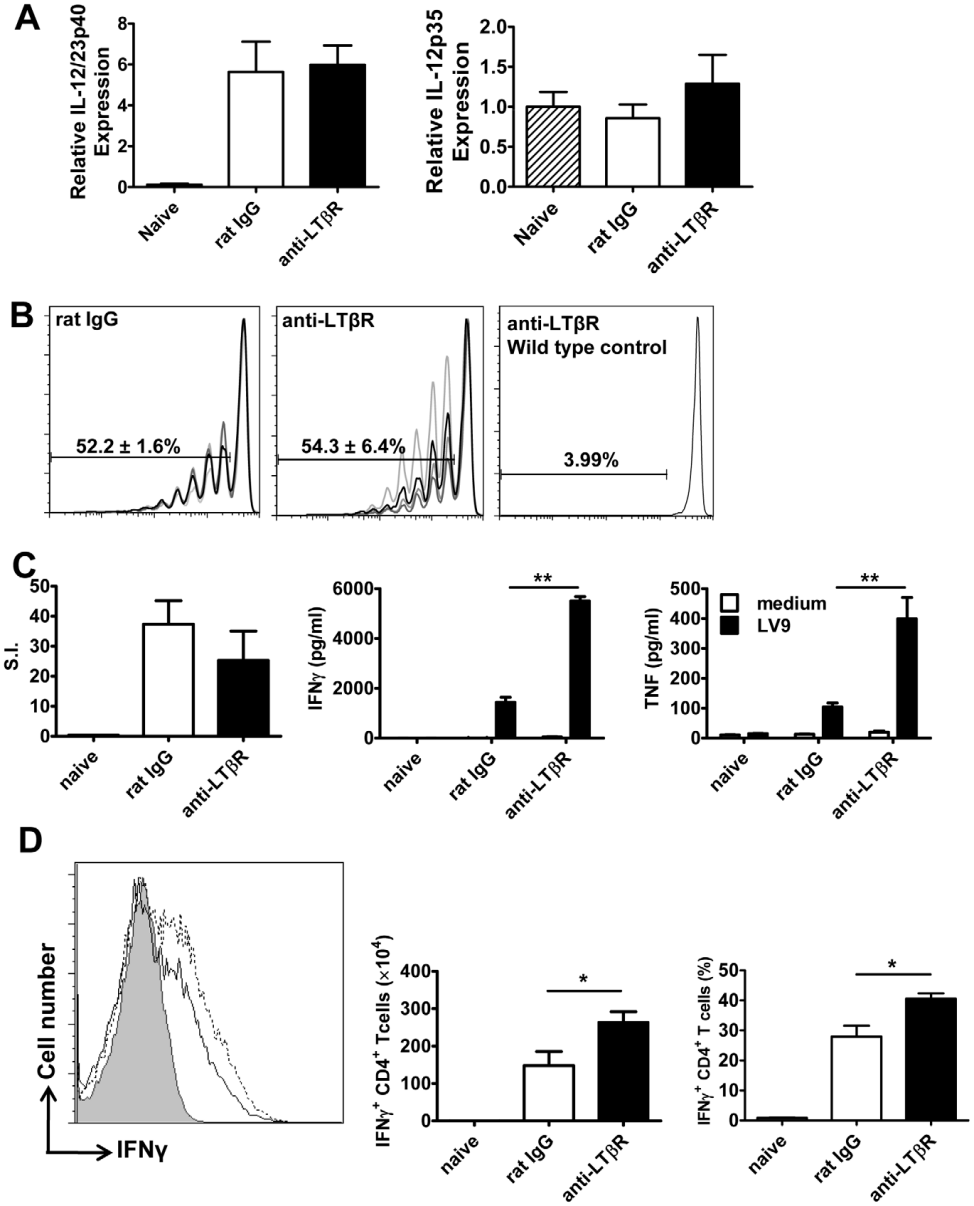
Treatment with anti-LTβR mAb LLTB2 leads to increased antigen-specific CD4^+^ T cell IFNγ and TNF production. DC IL-12/IL-23p40 mRNA accumulation (A), splenic OT-II CFSE dilution (B), antigen-specific splenic CD4^+^ T cell proliferation, IFNγ and TNF production (C) and hepatic CD4^+^ T cell IFNγ production (D) were measured as described in the legend of Figure 5. One representative experiment of 3 performed with similar outcome is shown (n = 4 mice per treatment group in each experiment). Statistical differences of p<0.05 (*), p<0.01 (**) or p<0.001 (***) for control versus mAb-treated mice are shown.

The anti-LTβR mAb (LLTB2) inhibited parasite growth in acute experimental VL (Figure 3A), but had no effect on splenic DC IL-12/IL-23p40 or IL-12p35 mRNA accumulation at 5 hours p.i. (Figure 6A), and no effect on the expansion of OVA-specific CD4^+^ T cells (OT-II cells) in mice infected with OVA-transgenic *L. donovani* (Figure 6B). We also found no differences in antigen-specific recall responses in splenic CD4^+^ T cells isolated from infected mice on day 14 p.i., yet the amount of TNF and IFNγ produced upon antigen-specific CD4^+^ T cell stimulation was greatly enhanced in these cells from mice treated with anti-LTβR mAb (Figure 6C). In addition, the number and frequency of IFNγ-producing hepatic CD4^+^ T cells measured directly *ex vivo* on day 14 p.i. was significantly increased in these mice (Figure 6D), suggesting LIGHT-LTβR binding suppresses the development of Th1 cell responses in VL.

### Treatment with anti- LTβR mAb promotes parasite clearance in the liver early during infection

**Figure 7:**
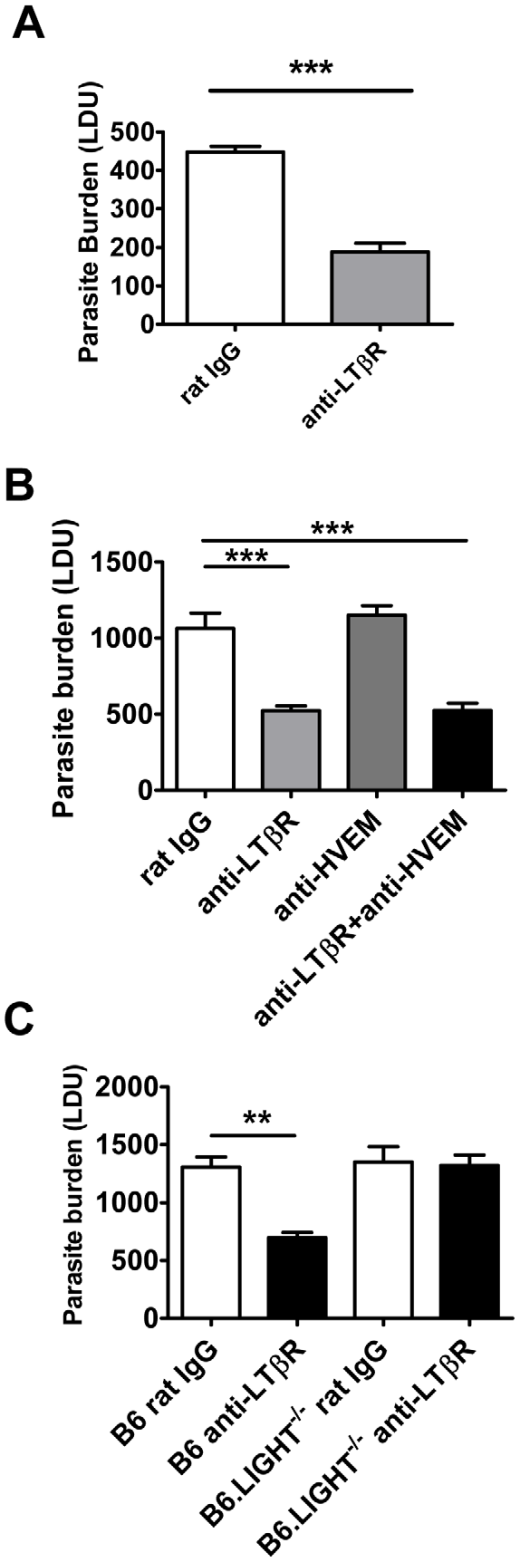
Blockade of LIGHT-LTβR signalling has an anti-parasitic effect at the time of infection in the liver. C57BL/6 mice were treated with anti-LTβR mAb (LLTB2) (light grey bars) or control rat IgG (open bars) and infected with L. donovani. Mice were injected with anti-LTβR mAb on the day of infection only and hepatic parasite burdens were determined in the liver at day 7 p.i. (A). C57BL/6 mice were treated with control rat IgG (open bars), anti-LTβR mAb (light grey bars), anti-HVEM mAb (dark grey bars) or both anti-LTβR and anti-HVEM mAbs (closed bars) on the day of L. donovani infection, and hepatic parasite burdens were determined in the liver at day 7 p.i. (B). C57BL/6 and B6.LIGHT^−/−^ mice were treated with anti-LTβR mAb (closed bars) or control rat IgG (open bars) on the day of L. donovani infection, and hepatic parasite burdens were determined in the liver at day 7 p.i. (C). Data are representative of two experiments performed (n = 5 mice per treatment group in each experiment). Statistical differences of p<0.01 (**) and p<0.001 (***) for control versus treated mice are shown.

We next investigated timing requirements for treatment with the anti-LTβR mAb (LLTB2) during acute infection with *L. donovani*. A single dose (100 µg) of anti-LTβR mAb at the time of infection was sufficient to reduce hepatic parasite burden as early as day 7 p.i. (Figure 7A). To test whether treatment with anti-LTβR mAb was simply shunting available LIGHT onto HVEM, we also co-treated mice with anti-LTβR (LLTB2) and anti-HVEM (LH1) mAbs (Figure 7B), and found no additional effect of co-administration over anti-LTβR alone by day 7 p.i., indicating that increased, early anti-parasitic immunity observed after anti-LTβR mAb (LLTB2) treatment was not caused by enhanced HVEM-mediated co-stimulation. Of note, there was no effect of anti-HVEM mAb treatment alone at day 7 p.i., indicating that the effect of this treatment on parasite burden only becomes apparent between days 7–14 p.i., similar to what was observed in LIGHT-deficient mice (Figure 1C). To test whether anti-LTβR mAb (LLTB2) agonist activity might account for the above effect, we treated LIGHT-deficient mice with this antibody and measured liver parasite burdens at day 7 p.i. (Figure 7C). Although a significant reduction in parasite burden was found in C57BL/6 mice treated with anti-LTβR mAb (LLTB2), no such effect was observed in LIGHT-deficient mice, indicating that the likely mechanism of action was via the blockade of LIGHT binding LTβR.

### Enhanced parasite clearance observed following treatment with anti-LTβR mAb requires CD4^+^ T cells and TNF

**Figure 8:**
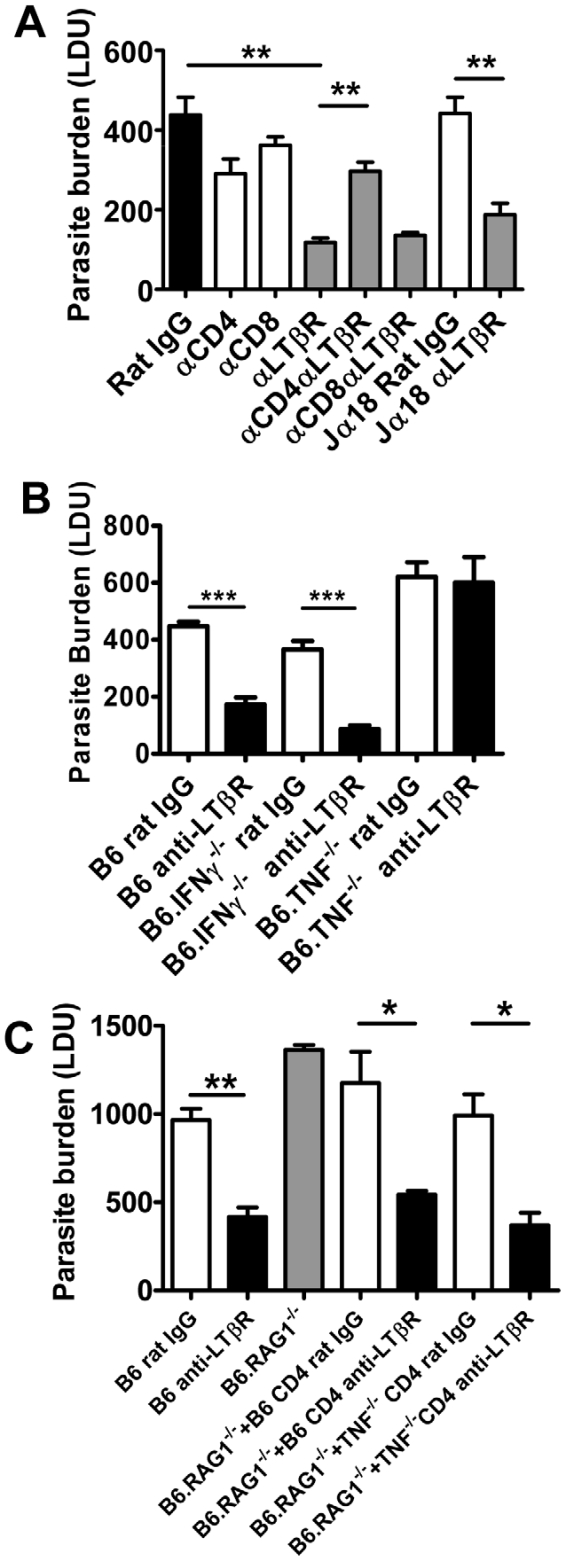
CD4^+^ T cells and TNF are required for enhanced parasite clearance following treatment with anti-LTβR mAb LLTB2. (A) C57BL/6 mice and B6.Jα18^−/−^ mice were treated with control rat IgG or anti-LTβR mAb on the day of L. donovani infection, with or without CD4^+^ or CD8^+^ T cell depletion, as indicated. Hepatic parasite burdens were determined at day 7 p.i.. (B) C57BL6 mice, B6.TNF^−/−^ mice and B6.IFNγ^−/−^ mice were treated with control rat IgG (open bars) or anti-LTβR mAb (LLTB2) (closed bars) on the day of infection with L. donovani and hepatic parasite burdens were determined at day 7 p.i.. (C) C57BL/6 mice and B6.RAG1^−/−^ mice were treated with control rat IgG (open bars) or anti-LTβR mAb on the day of L. donovani infection, as indicated. Prior to infection, B6.RAG1^−/−^ mice also received splenic CD4^+^ T cells from C57BL/6 mice or B6.TNF^−/−^ mice, as indicated. Hepatic parasite burdens were measured at day 7 p.i.. Data are from one of two experiments performed (n = 5 mice per treatment group in each experiment). Statistical differences of p<0.05 (*), p<0.01 (**) or p<0.001 (***) for control versus treated mice are shown.

We investigated the cellular requirements for the early anti-parasitic effects of anti-LTβR mAb (LLTB2). Treatment with anti-LTβR mAb had no impact on hepatic parasite burdens in B6.RAG1^−/−^ mice at day 7 p.i. (Figure S3), suggesting that B and/or T lymphocytes are required for the enhanced parasite clearance resulting from this treatment. We focused our attention on T cells because we have previously shown that B cells play a negative regulatory role in the liver during infection [43]. Depletion of CD4^+^ or CD8^+^ T cells alone during the first 7 days of infection had no effect on hepatic parasite burden (Figure 8A), despite T cells being required for the control of parasite growth at later stages of infection [26], [44], [45]. However, depletion of CD4^+^ T cells, but not CD8^+^ T cells, prevented the anti-parasitic effect mediated by anti-LTβR at day 7 p.i. (Figure 8A). Given that NKT cells comprise a significant proportion of hepatic CD4^+^ T cells, we also investigated whether this cell subset was required for the increased anti-parasitic activity. Treatment of NKT cell-deficient (B6.Jα18^−/−^) mice with the anti-LTβR mAb (Figure 8A) had no impact on the decreased liver parasite burden, indicating that conventional CD4^+^ T cells, but not NKT cells, are required for the enhanced parasite clearance following anti-LTβR mAb treatment.

We observed increased CD4^+^ T cell TNF and IFNγ production was associated with improved control of parasite growth resulting from anti-LTβR mAb treatment (Figure 6). We next assessed whether these cytokines were required for the enhanced parasite clearance in mice receiving anti-LTβR (LLTB2) mAb. Hepatic parasite burdens were decreased similarly in anti-LTβR mAb treated control and IFNγ-deficient mice (Figure 8B). However, anti-LTβR mAb treatment in TNF-deficient mice had no impact on hepatic parasite burden (Figure 8B), indicating that TNF is critical for this enhanced parasite clearance. The failure of anti-LTβR mAb treatment in TNF-deficient animals was not caused by reduced expression of LTβR on the cells of these mice, as LTβR expression levels were no different to those on immune cells from C57BL/6 control mice (data not shown). Furthermore, adoptive transfer of wild type and TNF-deficient CD4^+^ T cells into B6.RAG1^−/−^ mice and treatment with anti-LTβR mAb demonstrated that CD4^+^ T cells did not have to produce TNF (Figure 8C). Thus, anti-LTβR mAb treatment increased early hepatic anti-parasitic immunity by mechanisms requiring conventional CD4^+^ T cells and TNF, the latter potentially coming from a non-T cell source.

## Discussion

We have identified distinct and opposing roles for LIGHT and its receptors during infection. LIGHT has important roles in T cell costimulation [3], [14]. Blockade of LIGHT impairs allogeneic T cell responses and graft versus host disease [46], [47], while over-expression of LIGHT by T cells causes inflammatory disease of the gut and reproductive tissues [48], [49]. Our results indicate that these effects could be mediated via the LIGHT-HVEM axis between T cells and DC. Early DC IL-12 production depends on the presence of T cells, and this IL-12 production is critical for generating anti-parasitic immune mechanisms that control *L. donovani* growth [24], [39], [40], [50]. Our finding that anti- HVEM mAb blocks IL-12/IL-23p40 mRNA accumulation during infection is consistent with a previous study that reported BM-derived DCs from LIGHT-deficient animals were impaired in their ability to produce IL-12 following activation *in vitro* [51]. This study also showed that blockade of LIGHT with soluble receptors in mice infected with *L. major*, a cause of cutaneous leishmaniasis, resulted in reduced IL-12 generation, associated with diminished CD4^+^ T cell IFNγ production and increased parasite growth and disease. Our finding that T cells did not have to produce LIGHT in order to promote anti-parasitic immunity, together with data from *L. major* infection in mice [51], support a model whereby DC-derived LIGHT interacts with T cell HVEM to promote DC IL-12 production.

The defect in anti-parasitic immunity observed in the absence of LIGHT was restricted to the liver, and not the spleen. The reason for this is unclear, but could relate to the requirement for cellular recruitment and granuloma development for control of parasite growth in the liver. Although increased tissue weight and cellular expansion are features of *L. donovani* infection in the spleen, organised inflammatory granulomas are rarely observed in this tissue [24], [28], [29], [30]. Importantly, parasite growth is contained in the spleen after 1–2 months of infection rather than efficiently controlled, as occurs in the liver. Therefore, it is possible that different anti-parasitic immune mechanisms operate in these two tissue sites during experimental VL with different requirements for LIGHT.

**Figure 9:**
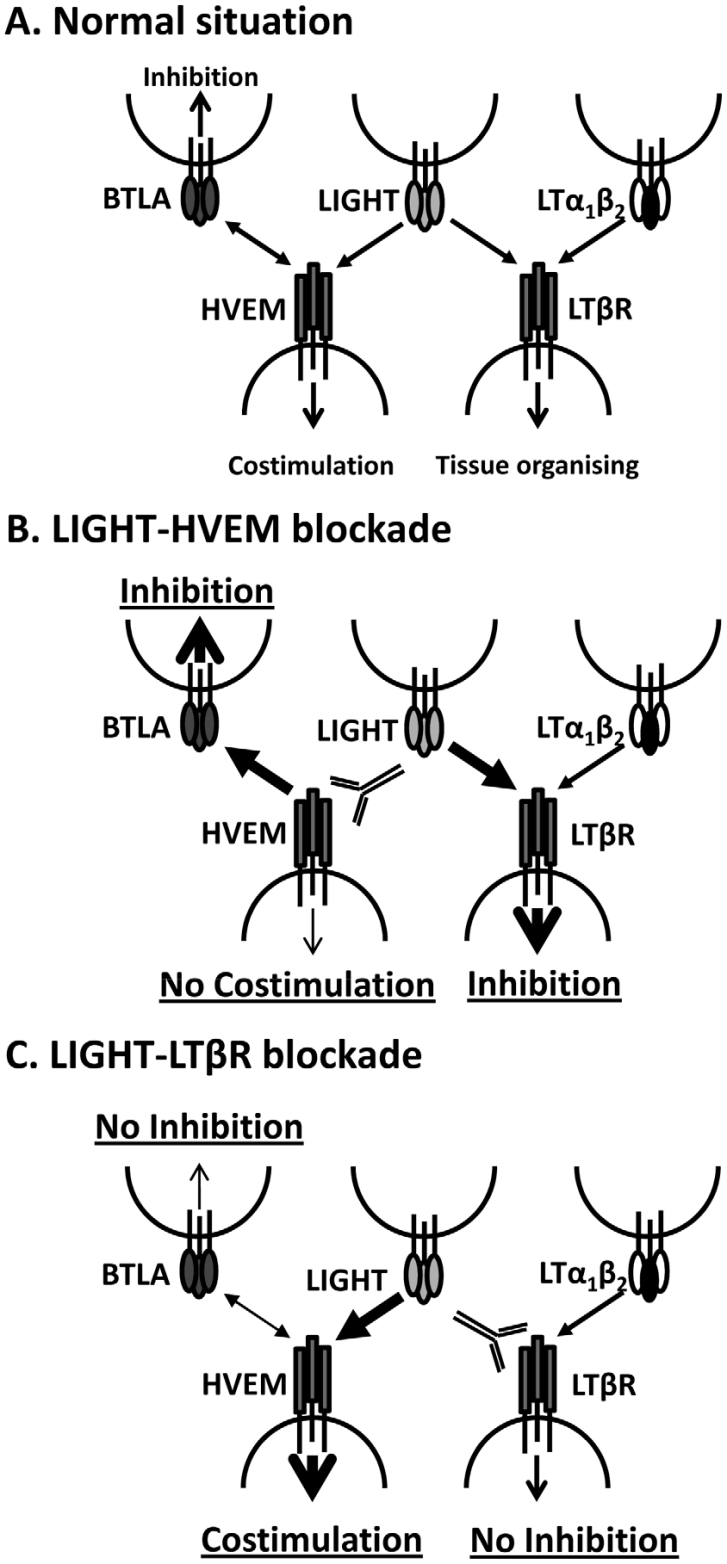
Model of how manipulation of interactions between LIGHT and its receptors may impact on disease outcome. A. Under normal circumstances LIGHT will interact with HVEM and/or LTβR to promote anti-parasitic immunity, as well as with DCR3 (not shown). HVEM can also interact with BTLA to suppress immune responses, and can also interact with CD160 and HSV1 gD (not shown). LTα_1_β_2_ can also bind to LTβR and provide signals essential for tissue organising and homeostasis. B. Blockade of LIGHT-HVEM with antibody will prevent HVEM-mediated costimulation and increase HVEM-BTLA-mediated inhibitory signals. LTβR-mediated inhibitory signals may also be increased via greater interactions with LIGHT. Together, this results in suppression of anti-parasitic immunity. C. Blockade of LIGHT-LTβR with antibody will potentially prevent LTβR-mediated inhibitory signals, but will also promote LIGHT-HVEM co-stimulatory signalling and reduce HVEM-BLTA inhibitory signals. Together, this stimulates increased anti-parasitic immunity.

The LIGHT-specific blocking mAbs we have employed (LH1 and LLTB2) have previously been shown to selectively block interactions between LIGHT and its receptors (HVEM and LTβR, respectively) [35]. However, we cannot exclude the possibility that they may trigger some receptor activation following engagement, and that this may contribute to biological effects we have observed. In addition, because these mAbs cause the selective blockade of LIGHT binding to their respective receptors, we cannot rule out that they promote alternative receptor-ligand interactions (Figure 9A). For example, blocking LIGHT interacting with HVEM may allow HVEM to more readily engage BTLA on cells to increase inhibitory signals (Figure 9B), as well as increased CD160 signalling. Similarly, blockade of LIGHT binding LTβR may allow greater amounts of LIGHT to bind HVEM, thereby reducing negative signalling between HVEM and BTLA and potentially promoting LIGHT-HVEM-mediated T cell co-stimulation (Figure 9C). However, this latter possibility seems unlikely in the current study given that co-administration of LH1 and LLTB2 resulted in improved control of parasite growth (Figure 7B). Instead, LIGHT may send inhibitory signals via LTβR early during infection, although no such LTβR-mediated negative signalling pathway has yet been defined.

The anti-parasitic effects of anti-LTβR mAb (LLTB2) were observed when it was administered at the time of infection, but not in mice with an established *L. donovani* infection, suggesting that a mAb with similar functional characteristics would have limited therapeutic potential for treatment of VL. However, our finding that the defined agonist anti-LTβR mAb (3C8) improved the rate of parasite clearance in the liver and reduced parasite load in the spleen, not only demonstrated fundamentally different biological activities for LLTB2 and 3C8 mAbs, but also shows that LTβR activation can promote beneficial immune mechanisms during established infection. This agonist antibody has previously been shown to promote DC development and maturation *in vivo* [20], [37], and this may explain the anti-parasitic effects observed after administration to *L. donovani*-infected mice because we have previously shown that DC adoptive transfer can improve control of parasite growth in infected mice [32]. Hence, the activation of anti-parasitic immune mechanisms by stimulation of LTβR represents a potential therapeutic strategy against chronic infectious diseases like VL. However, a better understanding of the functional characteristics of the different anti-LTβR mAbs will be required in order to better harness their therapeutic potential, including identification of the specific epitopes they recognise and signalling pathways they activate.

We previously reported increased monocyte recruitment into the spleen in an experimental model of cerebral malaria following treatment of mice with the anti-LTβR mAb (LLTB2), and that this treatment protected mice from disease [52]. Interestingly, no protection from experimental cerebral malaria was afforded by treatment with the anti-LTβR (3C8) mAb (Randall and Engwerda, unpublished), again emphasising the functional differences between LLTB2 and 3C8 anti-LTβR mAbs. An intriguing finding from our current studies was an increase in hepatic and splenic monocyte recruitment following anti-LTβR mAb LLTB2 treatment (CD11b^+^ Ly6C^hi^ cells; Figure S4A and B). Flow cytometry analysis revealed that monocytes, along with DCs (both cDC and pDC), and neutrophils expressed the highest levels of LTβR in the liver, as previously reported [19], [20], [37], and furthermore, that expression of LTβR did not appear to change significantly on any of these cells during the first 5 days of infection with L. donovani (Figure S5). However, the increased monocyte recruitment was not necessary for improved early control of parasite growth in treated animals in the current study because mice lacking CCL2 that have an impairment in monocyte mobilisation [53], also had improved control of parasite growth following anti-LTβR (LLTB2) treatment at day 7 p.i. (Figure S4C). Although the early anti-parasitic effect of anti-LTβR (LLTB2) mAb appeared to involve blocking of LIGHT- LTβR interactions, as indicated by the failure of this antibody to improve parasite control in LIGHT-deficient mice (Figure 7C), we cannot exclude the possibility that some effects of this antibody, such as increased monocyte mobilisation, might involve agonist activities. Regardless, given the important role for monocyte infiltration into sites of infection and tumour growth [54], our results indicate that manipulation of the LIGHT-LTβR signalling axis offers a potential way to improve monocyte mobilisation for therapeutic applications. Furthermore, given the recent report that monocytes can migrate into secondary lymphoid tissues in response to interactions with gram negative bacteria and/or their products, and then develop into CD209a^+^, CD206^+^, CD14^+^, CD11c^hi^ DC capable of activating CD4^+^ T cells and cross-priming CD8^+^ T cells [55], our results suggest that manipulation of LIGHT signalling pathways may be one way to promote this process that may have applications in vaccination.

In summary, our findings further delineate the complex interactions between LIGHT and its receptors and demonstrate the therapeutic potential of modulating these immune regulatory pathways to improve disease outcomes. Our results provide mechanistic insight into the roles of LIGHT-HVEM interactions on DC function and CD4^+^ T cell priming, as well as anti-parasitic immune responses activated by blockade of LIGHT-LTβR interactions. Finally, we have identified two different mAbs that target LTβR with distinct functional outcomes on anti-parasitic immunity at different stages of infection.

## Material and Methods

### Mice

Inbred female C57BL/6 and B6.SJL.*Ptprca* (B6.CD45.1) mice were purchased from the Australian Resource Centre (Canning Vale, Western Australia), and maintained under conventional conditions. B6.RAG1^−/−^ [56], B6.LIGHT^−/−^ [57], B6.TNF^−/−^ [58], B6.SJL.*Ptprca*×OT-II [59], B6.SJL.*Ptprca*×OT-I [60], B6.IFNγ^−/−^ [61] and B6.Jα18^−/−^ [62] were bred and maintained at the Queensland Institute of Medical Research. B6.CCL2^−/−^ mice [53] were bred at Monash University and maintained at the Queensland Institute of Medical Research. All mice used were age- and sex-matched (6–10 weeks), and were housed under specific-pathogen free conditions. Chimeric mice were prepared by irradiating B6.SJL.*Ptprca* mice with 11Gy and then engrafting with 3×10^6^ fresh bone marrow (BM) cells i.v. via the lateral tail vein. Mice were maintained on antibiotics for 2 weeks after engraftment and infected with *L. donovani* 8 weeks after receiving BM, as previously described [26]. Adoptive transfer of equal numbers (10^6^) of purified CD4^+^ and CD8^+^ T cells (98% purity as determined by flow cytometry) into B6.RAG1^−/−^ mice was performed as previously described [26].

### Ethics statement

All animal procedures were approved and monitored by the Queensland Institute of Medical Research Animal Ethics Committee. This work was conducted under QIMR animal ethics approval number A02-634M, in accordance with the “Australian code of practice for the care and use of animals for scientific purposes” (Australian National Health & Medical Research Council).

### Parasites and infections

*L. donovani* (LV9) and OVA-transgenic LV9 (PINK LV9) [42] were maintained by passage in B6.RAG1^−/−^ mice and amastigotes were isolated from the spleens of chronically infected mice. Mice were infected by injecting 2×10^7^ amastigotes i.v. via the lateral tail vein, killed at the times indicated in the text by CO_2_ asphyxiation and bled via cardiac puncture. In experiments examining DC IL-12/IL-23p40 production, mice were infected with 1×10^8^ amastigotes intravenously, as previously described [63]. Spleens and perfused livers were removed at times indicated and parasite burdens were determined from Diff-Quik-stained impression smears (Lab Aids, Narrabeen, Australia) and expressed as Leishman-Donovan units (LDU) (the number of amastigotes per 1,000 host nuclei multiplied by the organ weight in grams) [64]. Liver and spleen tissue were also preserved in either RNA*later* (Sigma-Aldrich, Castle Hill, Australia) or Tissue-Tek O.C.T. compound (Sakura, Torrence, USA). Hepatic mononuclear cells and splenocytes were isolated as previously described [65].

### Antibodies

All antibody-producing hybridomas were grown in 5% (v/v) foetal calf serum, RPMI containing 10 mM L-glutamine, 100 U/ml penicillin and 100 µg/ml streptomycin. Purified antibody was prepared as previously described [63]. Mice were administered 100 µg of anti-LTβR mAb (LLTB2) or anti-HVEM mAb (LH1) [35] i.v. on the day of infection and every 5 days thereafter for 14 day experiments, or as a single dose on the day of infection for 7 day experiments.. The anti-LTβR mAb 3C8 was administered at 200 µg i.v. [36], [37] starting at the times indicated in the text and every 5 days thereafter. The anti-LTβR mAb (LLTB2) or anti-HVEM mAb (LH1) specifically block the binding of LIGHT to either LTβR or HVEM, respectively, but do not disrupt interactions between these receptors and other functional ligands (i.e., LTα_1_β_2_ for LTβR and BTLA for HVEM) [35]. The anti-LTβR mAb (3C8) blocks binding of both LTα_1_β_2_ and LIGHT, but is an agonist directly activating LTβR [66]. Mice were depleted of CD4^+^ or CD8^+^ T cells with anti-CD4 (YTS191.1) or anti-CD8β (53-5.8) mAbs, respectively, as previously described [64]. Depletion of T cell subsets was confirmed at completion of experiments by assessing T cell numbers in the spleen by flow cytometry. Greater than 95% of CD4^+^ and CD8^+^ T cells were depleted by antibody treatment. In all experiments, control mice received the same quantities of the appropriate control hamster IgG (UC8-1B9; ATCC, Manassas, VA) or control rat IgG (Sigma-Aldrich).

### CD4^+^ T cell proliferation

To assess antigen-specific T cell proliferation *in vivo*, mice were infected with OVA-transgenic PINK LV9 [42]. Splenic OVA-specific OT-II T cells were isolated and labelled with CFSE, as previously described [64]. CFSE-labelled OT-II cells (1×10^6^) were adoptively transferred into mice 2 h prior to infection with LV9 or PINK LV9. Expansion of CFSE^+^ cells in the spleen was monitored by FACS 4 days later. In all of these experiments, control animals were included that received the same number of CFSE-labelled OT-II cells, but were infected with wild-type parasites. No OT-II proliferation was ever observed in these animals. Re-stimulation assays for endogenous splenic CD4^+^ T cells were performed as previously described [63].

### Assessment of granuloma formation

The maturation of granulomas was scored around infected Kupffer cells in acetone-fixed liver sections as previously described [64], [65].

### Flow cytometry

Allophycocyanin (APC)-conjugated anti-TCRβ chain (H57-597), B220 (clone RA3-6B2), CD11c (clone N418), phycoerythrin (PE)-Cy5-conjugated anti-CD4 (GK1.5), PE-conjugated IFNγ (XMG1.2), CD8β (53-5.8), I-A^b^ (clone AF6-120.1), Ly6G (clone 1A8), CD45.1 (clone A20), CD45.2 (clone 104), rat IgG1 (RTK2071), fluorescein isothiocyanate (FITC)-conjugated CD19 (clone 6D5), BST2 (clone 120G8), Ly6C (clone AL-21) and biotinylated anti-NK1.1 (PK136), CD11b (clone M1/70), LTβR (3C8) were purchased from Biolegend (San Diego, CA) or BD Biosciences (Franklin Lakes, NJ). Biotinylated antibodies were detected with streptavidin conjugated alexa 488, PE or PE/Cy5 (Biolegend). Leukocyte populations were defined as follows; CD4^+^ T cells (CD4^+^, TCR^+^), CD8^+^ T cells (CD8^+^, TCR^+^), NKT cells (NK1.1^+^, TCR^+^), NK cells (NK1.1^+^, TCR^−^), B cells (B220^+^, CD19^+^), cDC (CD11c^hi^, MHCII^hi^, TCR^−^, B220^−^), pDC (CD11c^int^, MHCII^int^, 120G8^+^), monocytes (CD11b^+^, Ly6C^+^) and neutrophils (CD11b^+^, Ly6G^+^). The staining of cell surface antigens and intracellular cytokine staining was carried out as described previously [63]. FACS was performed on a FACSCalibur or a FACS Canto II (BD Biosciences), and data were analysed using FlowJo software (TreeStar, Oregon, USA). Serum and/or tissue culture supernatants were assessed for the presence of soluble cytokines using flexset bead array kits and a FACSArray plate reader (BD Biosciences) according to the manufacturers' instructions.

### Real Time PCR

RNA extraction and real-time RT-PCR was performed as previously described [63]. The number of IFNγ, TNF, NOS-2, LIGHT and hypoxanthine phosphoribosyltransferase (HPRT) cDNA molecules in each liver tissue sample were calculated using Platinum Sybr Green Master Mix (Invitrogen Life Technologies) [63]. Standard curves were generated with known amounts of cDNA for each gene, and the number of cytokine molecules per 1000 HPRT molecules in each sample was calculated. The number of IL-12/IL-23p40 and IL-12p35 cDNA molecules in each DC sample were calculated using Taqman Gene Expression Assays (Applied Biosystems). Relative quantitation of gene expression was performed using the relative standard curve method as described by Applied Biosystems. Briefly, standard curves were prepared for all target and endogenous control genes using an uninfected control sample. HPRT was used as the endogenous control. The amount of target gene or endogenous control in each sample was calculated from the appropriate standard curves. The target amount was then divided by the endogenous control amount to give the normalized target value. The average normalized values for the four naïve samples were used as the calibrator.

### Statistical analysis

Statistical differences between groups was determined using the Mann-Whitney *U* test using GraphPad Prism version 4.03 for Windows (GraphPad Software, San Diego, CA) and p<0.05 was considered statistically significant. The distribution of hepatic histological responses was compared using X^2^ analysis with Microsoft Excel software. All data are presented as the mean values plus or minus standard error unless otherwise stated.

## Supporting Information

Figure S1 A persistent *L. donovani* infection becomes established in the spleen (A). Reduced cytokine production in *L. donovani*-infected LIGHT-deficient mice. (B) Day 14 p.i. serum TNF and IFNγ levels in C57BL/6 (closed bars) and B6.LIGHT^−/−^ mice (open bars).The accumulation of NOS2 (C), TNF (D) and IFNγ (E) mRNA levels in naïve or day 14 p.i. C57BL/6 and B6.LIGHT^−/−^ mice was detected by real time RT-PCR and is expressed as mRNA molecules per 1000 HPRT molecules (left panels are from liver and right panels are from spleen). Data are from one of two experiments performed (n = 4–5 mice per treatment group in each experiment). Statistical differences of p<0.05 (*) for C57BL/6 versus B6.LIGHT^−/−^ mice are shown.
(TIF)

Figure S2 No significant effect of blocking LIGHT-HVEM and LIGHT-LTβR interactions on parasite growth in the spleen in the first 14 days of infection. Parasite burdens were determined in the spleens of L. donovani infected mice treated with anti-HVEM (LH1) mAb or control hamster IgG or anti-LTβR (LLBT2) mAb or control rat IgG. Data are represented as the mean +/− SEM at day 14 p.i.. No statistical differences for control versus mAb-treated mice were found.
(TIF)

Figure S3 The anti-parasitic effect of blocking LIGHT-LTβR interactions fails in B6.RAG1^−/−^ mice. C57BL/6 or B6.RAG1^−/−^ mice were treated with control rat IgG (open bars) or anti-LTβR mAb (closed bars) the day prior to L. donovani infection, and hepatic parasite burdens were measured at day 7 p.i. and are represented as the mean +/−SEM. Statistical differences of p<0.05 (*) for control versus treated mice are shown.
(TIF)

Figure S4 Enhanced recruitment of inflammatory monocytes to the liver in mice in which LIGHT-LTβR interactions are blocked. (A) FACS profiles of hepatic monocytes (CD11b^+^Ly6C^hi^) from *L. donovani*-infected C57BL/6 mice at day 7 p.i., following treatment with either control rat IgG or anti-LTβR mAb prior to infection are shown. (B) Total numbers of CD11b^+^Ly6C^hi^ cells were measured from the livers of infected C57BL/6 mice and CCL2-deficient mice treated with either control rat IgG (open bars) or anti-LTβR mAb (closed bars) at day 7 p.i.. (C) C57BL/6 mice and B6.CCL2^−/−^ mice were treated with either control rat IgG (open bars) or anti-LTβR (closed bars), prior to L. donovani infection and hepatic parasite burdens were measured at day 7 p.i.. One representative experiment of two performed is shown (n = 5 mice per treatment group in each experiment). Statistical differences of p<0.05 (*) or p<0.01 (**) for control versus treated mice are shown.
(TIF)

Figure S5 Mononuclear cells isolated from the livers of naïve and L. donovani-infected mice at day 5 p.i. were phenotyped, labelled with a mAb against LTβR and enumerated by flow cytometry. Representative histograms gated on appropriate populations are shown for isotype control (solid grey shading), naïve mice (black line) and at day 5 p.i. with L. donovani (grey line). Cells were identified as follows: B cells (B220^+^CD19^+^), cDC(CD11c^hi^MHCII^hi^), monocytes (CD11b^+^Ly6c^hi^), neutrophils (CD11b^+^Ly6c^int^), NK cells (NK1.1^+^TCRβ^−^), NKT cells (NK1.1^+^TCRβ^+^), pDC (CD11c^int^120G8^+^), CD4^+^ T cells (CD4^+^TCRβ^+^), CD8^+^ T cells (CD8^+^TCRβ^+^). Data presented is representative of 2 independent experiments.
(TIF)
