# Supplementary figures and images for: Critical Roles for LIGHT and Its Receptors in Generating T Cell-Mediated Immunity during Leishmania donovani Infection
Source: PLoS Pathog. 2011 Oct 6;7(10):e1002279. doi: 10.1371/journal.ppat.1002279 (PMC3188526; doi:10.1371/journal.ppat.1002279)

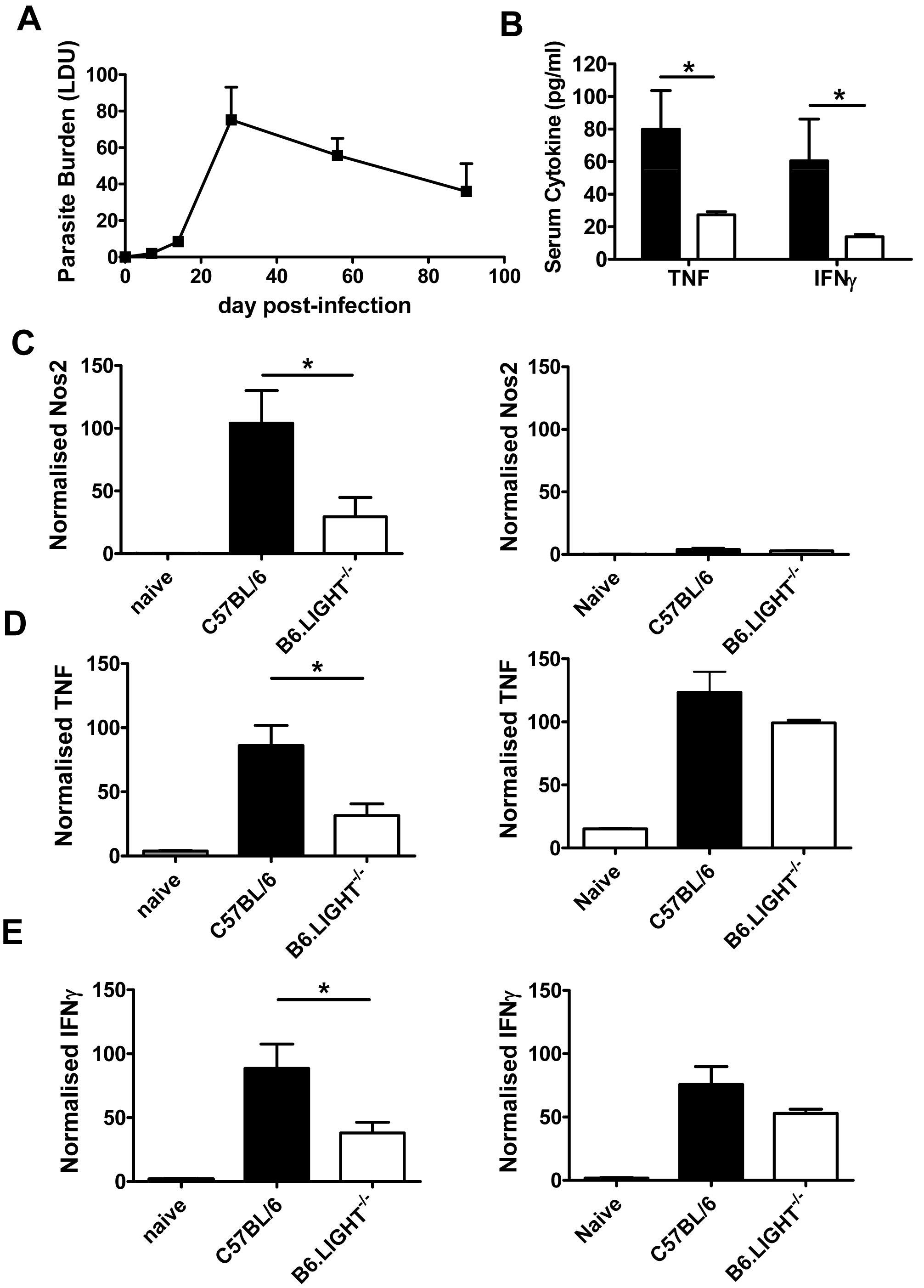

Supplement: Figure S1 — A persistent L. donovani infection becomes established in the spleen (A). Reduced cytokine production in L. donovani-infected LIGHT-deficient mice. (B) Day 14 p.i. serum TNF and IFNγ levels in C57BL/6 (closed bars) and B6.LIGHT−/− mice (open bars).The accumulation of NOS2 (C), TNF (D) and IFNγ (E) mRNA levels in naïve or day 14 p.i. C57BL/6 and B6.LIGHT−/− mice was detected by real time RT-PCR and is expressed as mRNA molecules per 1000 HPRT molecules (left panels are from liver and right panels are from spleen). Data are from one of two experiments performed (n = 4–5 mice per treatment group in each experiment). Statistical differences of p<0.05 (*) for C57BL/6 versus B6.LIGHT−/− mice are shown. (TIF) [file ppat.1002279.s001.tif]

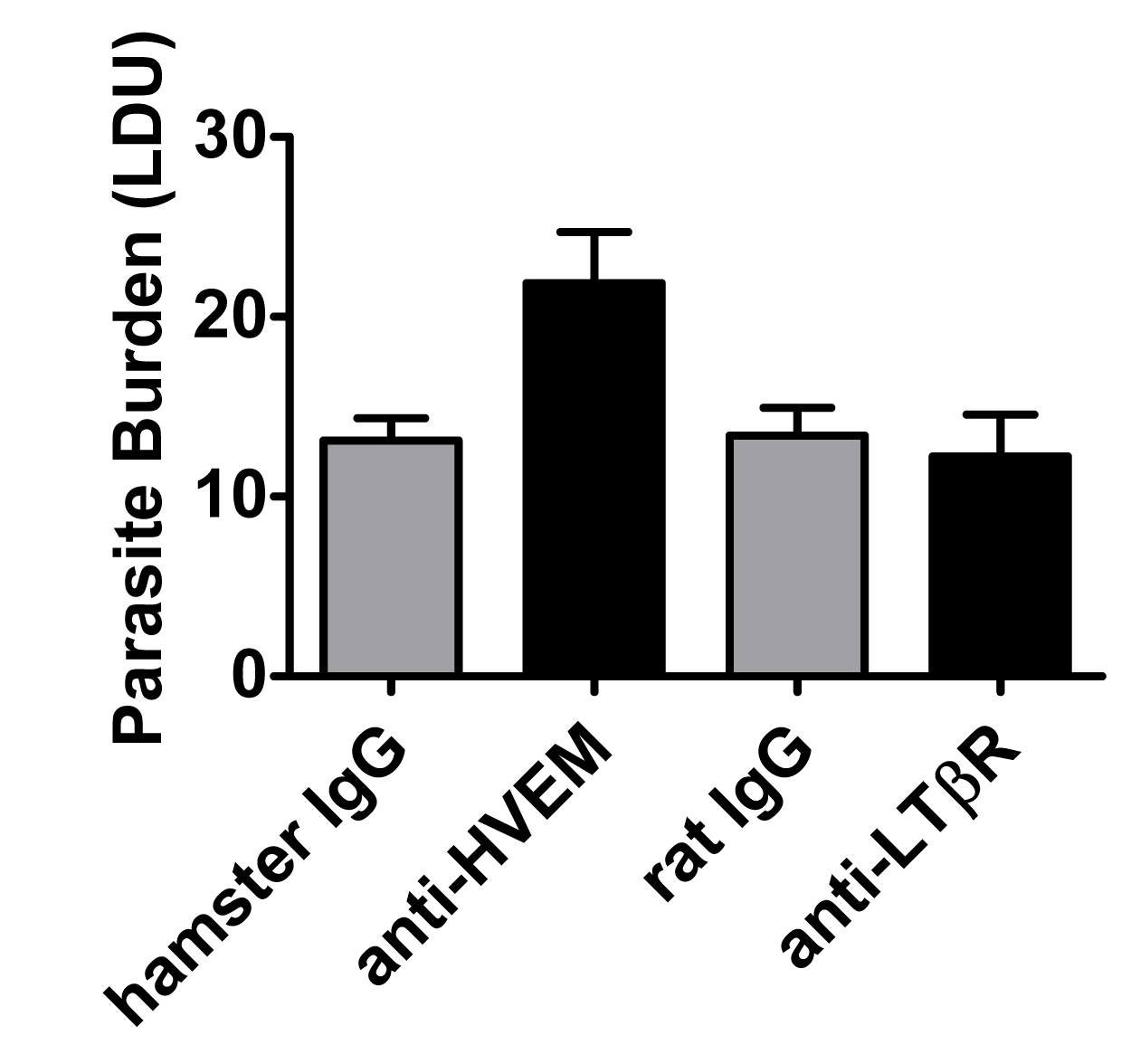

Supplement: Figure S2 — No significant effect of blocking LIGHT-HVEM and LIGHT-LTβR interactions on parasite growth in the spleen in the first 14 days of infection. Parasite burdens were determined in the spleens of L. donovani infected mice treated with anti-HVEM (LH1) mAb or control hamster IgG or anti-LTβR (LLBT2) mAb or control rat IgG. Data are represented as the mean +/− SEM at day 14 p.i.. No statistical differences for control versus mAb-treated mice were found. (TIF) [file ppat.1002279.s002.tif]

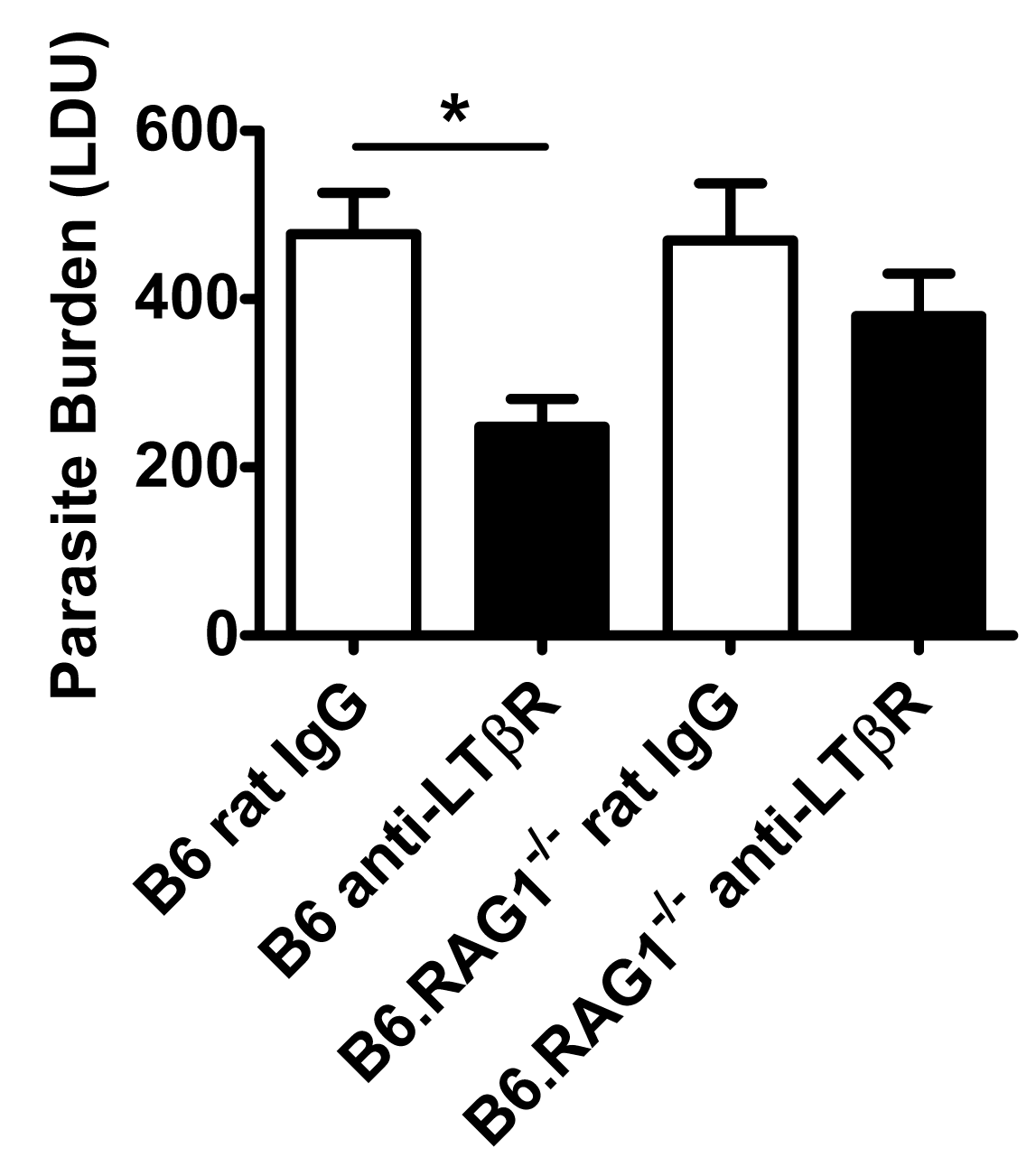

Supplement: Figure S3 — The anti-parasitic effect of blocking LIGHT-LTβR interactions fails in B6.RAG1−/− mice. C57BL/6 or B6.RAG1−/− mice were treated with control rat IgG (open bars) or anti-LTβR mAb (closed bars) the day prior to L. donovani infection, and hepatic parasite burdens were measured at day 7 p.i. and are represented as the mean +/−SEM. Statistical differences of p<0.05 (*) for control versus treated mice are shown. (TIF) [file ppat.1002279.s003.tif]

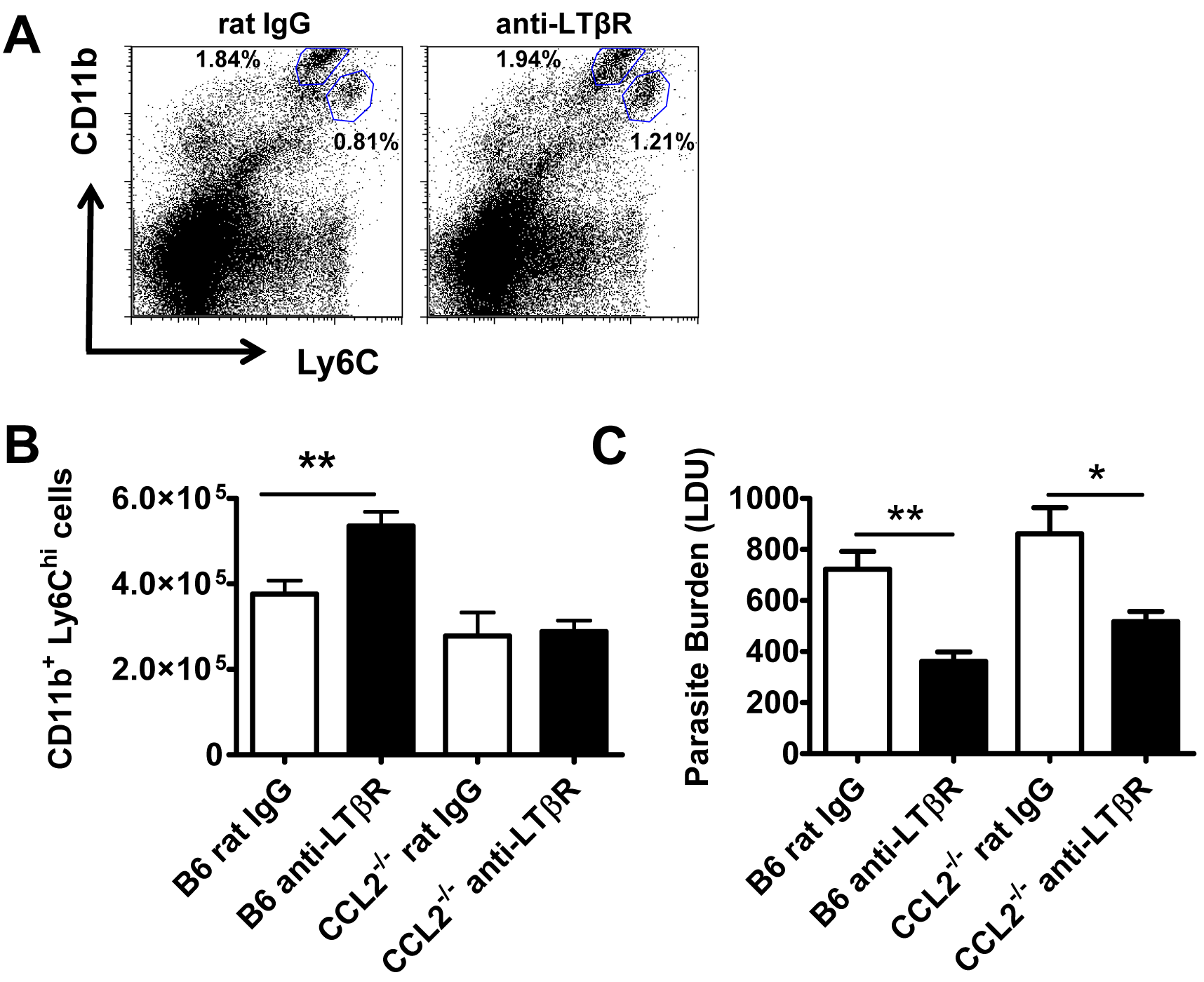

Supplement: Figure S4 — Enhanced recruitment of inflammatory monocytes to the liver in mice in which LIGHT-LTβR interactions are blocked. (A) FACS profiles of hepatic monocytes (CD11b+Ly6Chi) from L. donovani-infected C57BL/6 mice at day 7 p.i., following treatment with either control rat IgG or anti-LTβR mAb prior to infection are shown. (B) Total numbers of CD11b+Ly6Chi cells were measured from the livers of infected C57BL/6 mice and CCL2-deficient mice treated with either control rat IgG (open bars) or anti-LTβR mAb (closed bars) at day 7 p.i.. (C) C57BL/6 mice and B6.CCL2−/− mice were treated with either control rat IgG (open bars) or anti-LTβR (closed bars), prior to L. donovani infection and hepatic parasite burdens were measured at day 7 p.i.. One representative experiment of two performed is shown (n = 5 mice per treatment group in each experiment). Statistical differences of p<0.05 (*) or p<0.01 (**) for control versus treated mice are shown. (TIF) [file ppat.1002279.s004.tif]

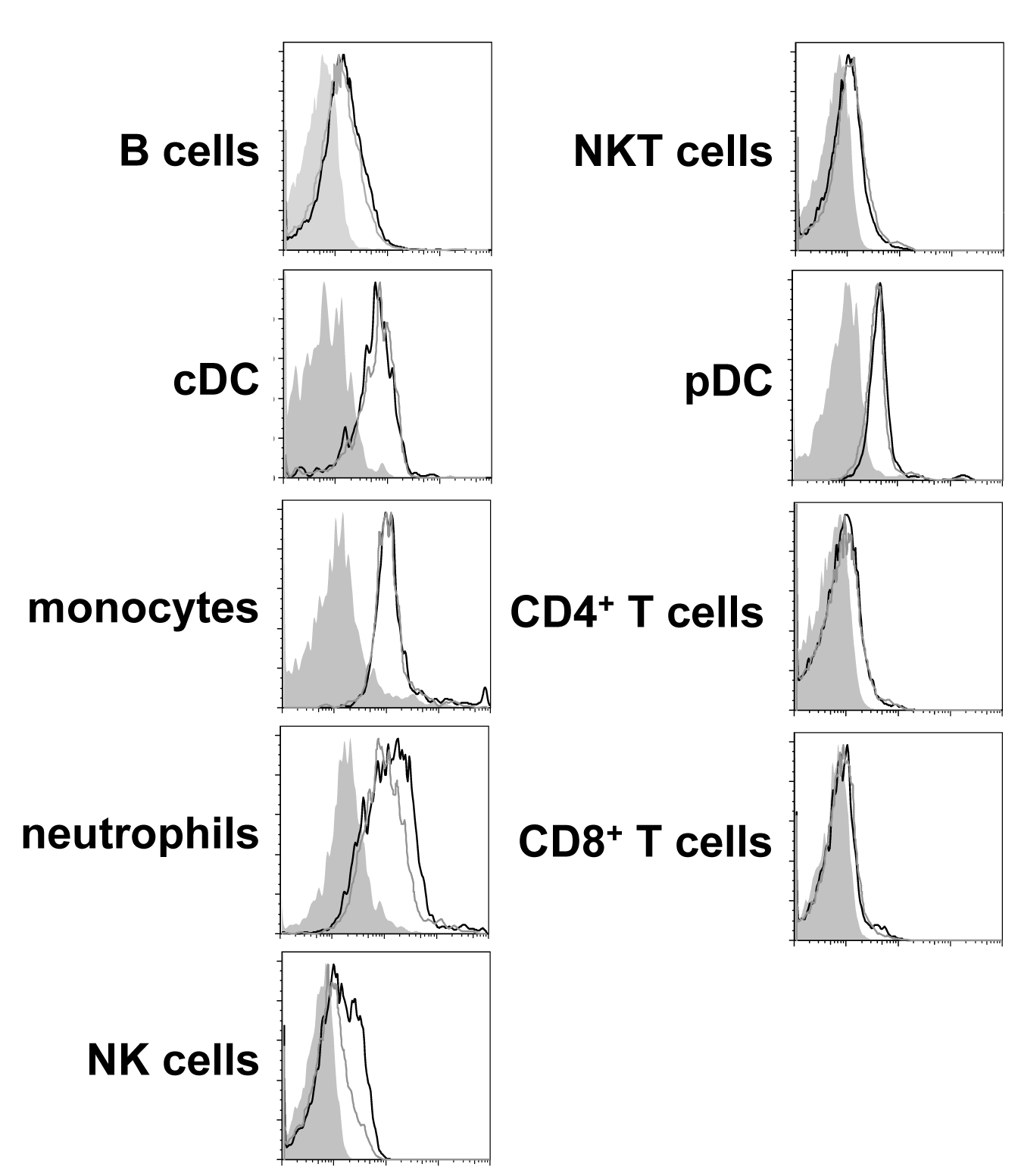

Supplement: Figure S5 — Mononuclear cells isolated from the livers of naïve and L. donovani-infected mice at day 5 p.i. were phenotyped, labelled with a mAb against LTβR and enumerated by flow cytometry. Representative histograms gated on appropriate populations are shown for isotype control (solid grey shading), naïve mice (black line) and at day 5 p.i. with L. donovani (grey line). Cells were identified as follows: B cells (B220+CD19+), cDC(CD11chiMHCIIhi), monocytes (CD11b+Ly6chi), neutrophils (CD11b+Ly6cint), NK cells (NK1.1+TCRβ−), NKT cells (NK1.1+TCRβ+), pDC (CD11cint120G8+), CD4+ T cells (CD4+TCRβ+), CD8+ T cells (CD8+TCRβ+). Data presented is representative of 2 independent experiments. (TIF) [file ppat.1002279.s005.tif]
